# Heterogeneous effect of aging on vasorelaxation responses in large and small arteries

**DOI:** 10.14814/phy2.14341

**Published:** 2020-01-21

**Authors:** Meredith Luttrell, Hyoseon Kim, Song Yi Shin, Dylan Holly, Michael P. Massett, Christopher R. Woodman

**Affiliations:** ^1^ Department of Health & Kinesiology Texas A&M University College Station Texas; ^2^ Department of Veterinary Physiology & Pharmacology Texas A&M University College Station Texas; ^3^ Present address: Department of Kinesiology and Sport Management Texas Tech University Lubbock Texas

**Keywords:** acetylcholine, calmodulin, caveolin‐1, endothelial nitric oxide synthase, sodium nitroprusside

## Abstract

Aging is associated with impaired vascular function characterized in part by attenuated vasorelaxation to acetylcholine (ACh) and sodium nitroprusside (SNP). Due to structural and functional differences between conduit and resistance arteries, the effect of aging on vasorelaxation responses may vary along the arterial tree. Our purpose was to determine age‐related differences in vasorelaxation responses in large and small arteries. Responses to the endothelium‐dependent vasodilator acetylcholine (ACh) and the endothelium‐independent vasodilator sodium nitroprusside (SNP) were assessed in abdominal aorta (AA), iliac arteries (IA), femoral arteries (FA), and gastrocnemius feed arteries (GFA) from young and old male rats. ACh‐mediated vasorelaxation was significantly impaired in old AA and IA. SNP‐mediated vasorelaxation was impaired in old AA. To investigate a potential mechanism for impaired relaxation responses in AA and IA, we assessed eNOS protein content and interactions with caveolin‐1 (Cav‐1), and calmodulin (CaM) via immunoprecipitation and immunoblot analysis. We found no age differences in eNOS content or interactions with Cav1 and CaM. Combined data from all rats revealed that eNOS content was higher in IA compared to AA and FA (*p* < .001), and was higher in GFA than AA (*p* < .05). Cav1:eNOS interaction was greater in FA than in AA and IA (*p* < .01), and in GFA compared to IA (*p* < .05). No differences in CaM:eNOS were detected. In conclusion, age‐related impairment of vasorelaxation responses occurred in the large conduit, but not small conduit or resistance arteries. These detrimental effects of age were not associated with changes in eNOS or its interactions with Cav‐1 or CaM.

## Graphical abstract

Our purpose was to determine age‐related differences in vasorelaxation responses in large and small arteries. Results revealed that age‐related impairment of vasorelaxation responses occurred in the large conduit, but not small conduit or resistance arteries. These detrimental effects of age were not associated with changes in eNOS or its interactions with Cav‐1 or CaM.

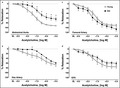

## 1 INTRODUCTION

The well‐documented age‐related decline in arterial vascular function independently increases the risk of developing cardiovascular diseases (Celermajer et al., 1994). Given the heterogeneity of arterial vascular structure and function throughout the arterial network (Aird, 2007), the effects of aging on endothelium‐dependent vasorelaxation responses to acetylcholine (ACh) and endothelium‐independent relaxation to sodium nitroprusside (SNP) may vary with vascular phenotype. Age‐related impairments in arterial vascular function have been demonstrated in the aorta (Barton et al., 1997; van der Loo et al., 2000), and in resistance‐sized arteries, including the middle cerebral artery (Walker et al., 2015), renal artery (Meyer, Rosemann, Barton, & Prossnitz, 2017), mesenteric artery (Boerman, Everhart, & Segal, 2016), and the soleus feed artery (Woodman, Price, & Laughlin, 2002). However, impaired arterial vascular function is not a universal finding with advanced age. Notably, endothelial function in the gastrocnemius feed artery (GFA) appears to be maintained with age (Woodman et al., 2002), while the effect of age on femoral artery vascular function is unclear. Barton et al. (1997). report preserved NO‐mediated endothelial function in the femoral artery. However, preserved endothelial function with age in the femoral artery has not been consistently established. Harris and colleagues reported impaired ACh‐mediated relaxation of the femoral artery in aged rats (Harris, Slack, Prestosa, & Hryvniak, 2010). Together, these studies suggest that while aging is associated with impaired vascular function in many arterial beds, some arteries may be protected from the detrimental effects of aging. Accordingly, the mechanisms accounting for the decline in vasorelaxation responses with age may be different in large conduit arteries, like the aorta and iliac artery, versus small conduit and resistance arteries, such as the femoral artery and GFA.

Age‐related changes in post‐translational mechanisms regulating endothelial nitric oxide synthase (eNOS) enzyme activity are an intriguing candidate as a potential mechanism accounting for impaired vasorelaxation responses to ACh with age. Acetylcholine stimulates receptor‐mediated activation of PI3K/Akt pathway, resulting in an increase in intracellular calcium, which binds to calmodulin (CaM). The calcium–CaM complex binds to and displaces eNOS from its binding site on caveolin‐1, and increases eNOS activity and NO production (Fleming & Busse, 1999). Numerous groups have shown that NO‐mediated relaxation is impaired as a consequence of aging in both conduit and resistance arteries and arterioles in animal models (Barton et al., 1997; Hirai, Copp, Hageman, Poole, & Musch, 2011; Soucy et al., 2006; Woodman, Price, & Laughlin, 2003) and in humans (Nyberg et al., 2012). This age‐related impairment in NO bioavailability has been partially attributed to post‐translational modifications of eNOS, resulting in reduced NO bioavailability (Fulton, Gratton, & Sessa, 2001). Post‐translational regulation of eNOS is complex, but includes protein modifications controlling catalytic activity and the availability of eNOS substrates and cofactors (Qian & Fulton, 2013). One post‐translational modification that may contribute to impaired ACh‐mediated NO production with age is an alteration in protein:protein interactions among eNOS and two of its key regulatory proteins, caveolin‐1 (Cav1) and calmodulin (CaM). Cav1, a protein localized in caveolae of the plasma membrane, is a negative regulator of eNOS activity (Michel, Feron, Sacks, & Michel, 1997), such that greater Cav1 binding to eNOS may limit NO bioavailability and result in impaired NO‐mediated endothelial function. In contrast, calcium‐sensitive CaM is a positive regulator of eNOS activity (Michel et al., 1997), thus potentially increasing enzyme activity and NO bioavailability when bound to eNOS. Luttrell, Seawright, Wilson, & Woodman (2013) showed previously that both Cav1:eNOS and CaM:eNOS interactions were blunted with aging in rat aortas, and that these changes were not altered with exercise training. However, the phenotype of these eNOS‐associated protein:protein interactions throughout the arterial network remains unclear.

The purpose of the present study was to (1) determine and compare the effect of age on ACh‐ and SNP‐mediated vasorelaxation in the abdominal aorta (AA), iliac artery (IA), femoral artery (FA), and gastrocnemius feed artery (GFA) and (2) determine eNOS‐associated protein:protein interactions in the AA, IA, FA, and GFA.

## 2 MATERIALS AND METHODS

### 2.1 Ethics approval

Prior to initiating this study, approval was received from the Texas A&M University Institutional Animal Care and Use Committee. All procedures were performed under the Public Health Service's Policy on Human Care and Use of Laboratory Animals guidelines. Young (4 months, *n* = 20) and old (24 months, *n* = 20) male Fischer 344 rats were obtained from the National Institute on Aging (NIA) and housed at the Texas A&M Comparative Medicine Program Facility. All rats were housed under a 12:12 hr light–dark cycle and food and water were provided ad libitum. The rats were examined daily by Animal Care Facility veterinarians or staff.

### 2.2 Isolation of arteries

Prior to removal of arteries, rats were anesthetized with an intraperitoneal injection of Ketamine (80 mg/kg body weight) and Xylazine (5 mg/kg body weight). The thoracic and abdominal cavities were exposed and the abdominal aorta (AA), iliac arteries (IA), and femoral arteries (FA) were dissected and placed in cold (4°C) MOPS‐buffered physiological saline solution (PSS), containing (in mM): 145.0 NaCl, 4.7 KCl, 2.0 CaCl_2_, 1.17 MgSO_4,_ 1.2 NaH_2_PO_4_, 5.0 glucose, 2.0 pyruvate, 0.02 EDTA, and 25.0 MOPS (pH 7.4). In addition, gastrocnemius muscle feed arteries (GFA) were removed from the left and right hindlimbs. GFA are resistance‐sized arteries responsible for providing blood flow to the highly glycolytic gastrocnemius muscle. These arteries were selected because they represent three functional phenotypes of arteries (large conduit, small conduit, and resistance‐sized arteries). These vessels are also all large enough to perform myography experiments as described below, allowing direct comparison of vasorelaxation responses using the same experimental preparation.

Rats were then euthanized by excision of the heart. Arteries were transferred to a Lucite chamber containing MOPS‐PSS for removal of adipose and connective tissue, and sectioned into 2 mm rings. Arterial rings used for the assessment of vasomotor function were mounted on a wire myograph. In a separate group of young and old rats, arteries were isolated and placed in 0.7 ml microcentrifuge tubes, snap frozen, and stored at −80°C for subsequent immunoprecipitation and immunoblot analysis.

### 2.3 Determination of vasorelaxation responses

Vasorelaxation responses were assessed as described previously (Kim, Avila, & Massett, 2016). Briefly**,** arterial ring segments were mounted in a wire myograph (Danish Myo Technology, Aarhus, Denmark) filled with 8 ml of oxygenated (95% O_2_, 5% CO_2_) physiological saline solution (in mM: 118.31 NaCl, 4.69 KCl, 1.2 MgSO_4_, 1.18 KH_2_PO_4_, 24.04 NaHCO_3_, 0.02 EDTA, 2.5 CaCl_2_, and 5.5 glucose). Rings were maintained at 37°C and pH 7.4 throughout the experiment. Optimal resting tension was determined for each ring segment using a standard normalization procedure and arterial rings were pre‐constricted to 70% of maximal contraction with phenylephrine (PE) (Angus, Cocks, & Satoh, 1986). After reaching stable tension, endothelium‐dependent relaxation responses were assessed by adding cumulative concentrations of ACh over the range of 10^−9^–10^−5^ M in half log increments. Endothelium‐independent relaxation responses were measured by adding cumulative concentrations of sodium nitroprusside (SNP) over the range of 10^−9^–10^−5^ M in half log increments. Percent relaxation responses were calculated as [(D_P_ − D_D_)/(D_P_ − D_B_)] × 100, where *D_P_* is the maximal force pre‐generated by PE, *D_D_* is the lowest force generated at a given dose of ACh or SNP, and *D_B_* is the baseline force.

### 2.4 Arterial homogenization

AA, IA, and FA were homogenized using a mortar and pestle, then transferred to a microcentrifuge tube with a lysis buffer containing (in mM) 60.0 octyl‐β‐d‐glucopyranoside (Sigma‐Aldrich, O8001), 150.0 NaCl, 20.0 Tris base, 1% protease inhibitor cocktail (EMD Millipore, 20‐201), and 1% phosphatase inhibitor cocktail (Sigma‐Aldrich, P0044). These vessel segments were further homogenized using repeated freeze–thaw cycles with brief sonication. For homogenization of the GFA, lysis buffer was added to the microcentrifuge tubes for homogenization using repeated freeze–thaw cycles. To collect the soluble proteins from all vessels, the homogenates were centrifuged for 10 min at 12,000*g* at 4°C and the supernatant was collected and used for immunoprecipitation.

### 2.5 Immunoprecipitation of eNOS

Immunoprecipitation of eNOS was performed as described previously (Luttrell et al., 2013). Twenty µl of Protein G/A agarose suspension (Millipore, IP05) was added to the artery lysates, incubated at 4°C for 1 hr under gentle rotary agitation, and centrifuged at 12,000*g* for 1 min. The supernatant was collected and 1 µg of polyclonal eNOS antibody (BD Transduction, 610298) was added to the lysate and incubated for 4 hr at 4°C under gentle rotary agitation. Protein G/A agarose suspension (15 μL) was then added to the lysate and incubated for an additional 1 hr at 4°C under gentle rotary agitation. The eNOS protein complex was then eluted from the lysate, washed three times and stored at −80°C in Laemmli buffer for immunoblotting.

### 2.6 Quantification of Cav1:eNOS and CaM:eNOS interactions

Total protein content was assessed using a bicinchoninic acid protein assay. Immunoprecipitated eNOS, and eNOS‐associated Cav1 and CaM protein content were evaluated using monoclonal antibodies (eNOS [Becton‐Dickson, 610297] and CaM [Invitrogen, 13‐6900], 1:1,250; Cav1 [Becton‐Dickson, 610406], 1:2,500). Immunoblots were evaluated using enhanced chemiluminescence (ECL, Amersham) and densitometry using LAS‐4000 Luminescent Image Analyzer and Multi‐Gauge Image Analysis Software (FUJIFILM Medical Systems).

### 2.7 Statistical analysis

All values are means ± *SD*. Concentration–response curves were analyzed by two‐way ANOVA with repeated measures on one factor (concentration). When a significant *F* value was obtained, post‐hoc analyses were performed using Duncan's multiple range test. The half‐maximal inhibitory concentrations (IC_50_) for ACh and SNP were calculated with absolute values from cumulative concentration–response curves to each agent, using the logarithmic dose–response with four‐parameter variable curve fitting algorithm in GraphPad Prism 6. The effect of age on the IC_50_ and the percent of maximal response to 10^−5^ M for ACh and SNP were assessed within each vessel using unpaired *t* tests. Statistical analysis for vasorelaxation responses was performed using GraphPad Prism6.

Immunoprecipitated eNOS protein content in all vessels was normalized to average immunoprecipitated eNOS protein content in AA based on densitometry values to show fold changes in immunoprecipitated eNOS protein among large and small arteries. Protein:protein interaction data are presented as a ratio of densitometry units for Cav1 to eNOS or CaM to eNOS for all vessels. Cav1:eNOS and CaM:eNOS ratios were normalized to the respective average ratio for AA to show fold changes in protein:protein interactions in the arteries studied. Un‐paired *t* tests were used to determine the effect of aging on immunoprecipitated eNOS protein and associated protein interactions within a single vessel. Because this analysis revealed no age‐group differences in immunoprecipiated eNOS protein or eNOS protein interactions in any artery studied, data from young and old rats were pooled to assess branch order differences. One‐way ANOVA was used to determine vessel‐group differences in total eNOS protein content and protein:protein interactions. Tukey's multiple comparisons test was used when a significant *F* value was obtained. Statistical significance was set at *p* ≤ .05 probability level. Statistical analyses for protein data were performed using GraphPad Prism7.04.

## 3 RESULTS

### 3.1 ACh‐induced relaxation

**Figure 1:**
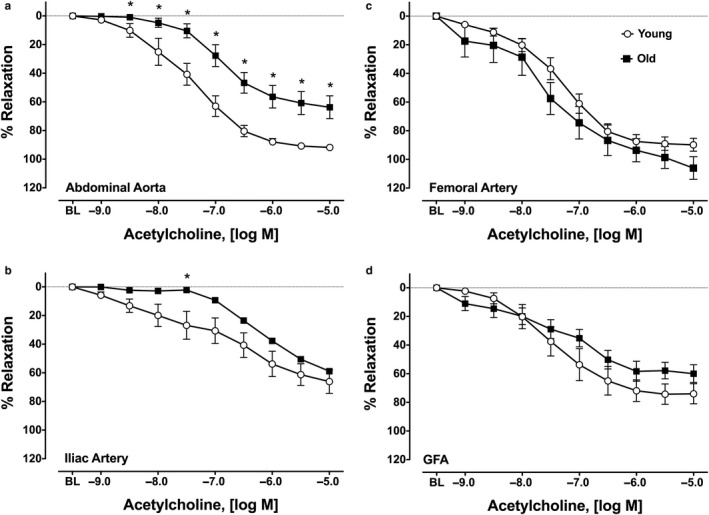
Acetylcholine (ACh)‐induced relaxation in abdominal aorta (a), iliac arteries (b), femoral arteries (c), and gastrocnemius feed arteries (d). Values are means ± *SD*; *n* = 6–9 per group. *BL*, baseline after 70% maximal contraction with phenylephrine. *Significantly different from young, *p* ≤ .05

ACh elicited a significant concentration‐dependent relaxation response in AA, IA, FA, and GFA (Figure 1a–d, respectively). ACh‐induced relaxation was significantly impaired in AA and IA from old rats relative to the young rats. Sensitivity (IC_50_) and % maximal relaxation at 10^−5^ M of ACh were significantly lower in the AA of old compared to young rats (*p* < .05), but was not altered with age in IA, FA, or GFA (Table 1). ACh‐induced relaxation was not impaired in old FA or GFA.

**Table 1:** IC_50_ –log *M* values and response to 10^−5^ M ACh and SNP from large and small arteries from young and old rats. Values are means ± *SD*; *n* = 6–9 per group. *Significantly different from young, *p* < .05

	Young		Old	
	IC50, −log M ACh	Response to 10^−5^ M ACh, % maximal relaxation	IC50, −log M SNP	Response to 10^−5^ M ACh, % maximal relaxation
AA	−7.46 ± 0.57	91.84 ± 3.79	−6.93 ± 0.42*	63.8 ± 22.51*
IA	−7.09 ± 1.27	66.13 ± 21.87	−6.20 ± 0.37	58.88 ± 15.03
FA	−7.29 ± 0.45	89.83 ± 13.55	−7.22 ± 0.88	106.09 ± 22.36
GFA	−7.35 ± 0.56	74.02 ± 18.54	−7.01 ± 0.68	59.98 ± 17.54

	IC50, −log M SNP	Response to 10^−5^ M SNP, % maximal relaxation	IC50, −log M SNP	Response to 10^−5^ M SNP, % maximal relaxation
AA	−7.83 ± 0.99	100 ± 1.35	−7.08 ± 0.85	96.21 ± 7.47
IA	−8.03 ± 0.54	95.32 ± 7.19	−7.31 ± 0.45^*^	107.01 ± 23.49
FA	−7.39 ± 1.26	100.85 ± 2.19	−6.66 ± 1.10	118.47 ± 17.89
GFA	−7.65 ± 0.54	99.35 ± 2.89	−6.72 ± 0.45	100.94 ± 25.72

### 3.2 SNP‐induced relaxation

**Figure 2:**
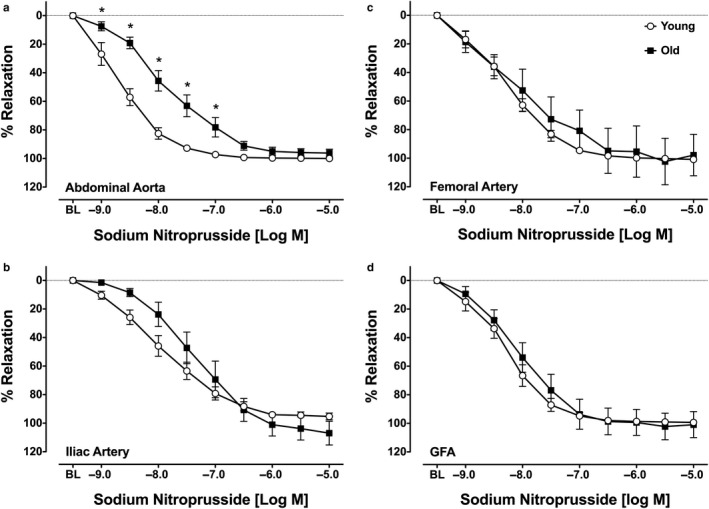
Sodium nitroprusside (SNP)‐induced relaxation in abdominal aorta (a), iliac arteries (b), femoral arteries (c), and gastrocnemius feed arteries (d). Values are means ± *SD*; *n* = 7–9 per group. *BL*, baseline after 70% maximal contraction with phenylephrine. *Significantly different from young, *p* ≤ .05

SNP elicited a significant relaxation response in the AA, IA, FA, and GFA (Figure 2a–d, respectively). SNP‐induced relaxation was significantly impaired in AA from old rats relative to the young rats. SNP‐induced relaxation was not impaired by age in IA, FA, or GFA. Sensitivity to SNP was significantly lower in the IA of old compared to young rats (*p* = .01), but was not altered with age in the AA, FA, or GFA (Table 1). Age had no effect on % maximal relaxation at 10^−5^ M SNP in any vessel.

### 3.3 Immunoprecipitated eNOS and eNOS‐associated protein:protein interactions in arteries from young and older rats

**Figure 3:**
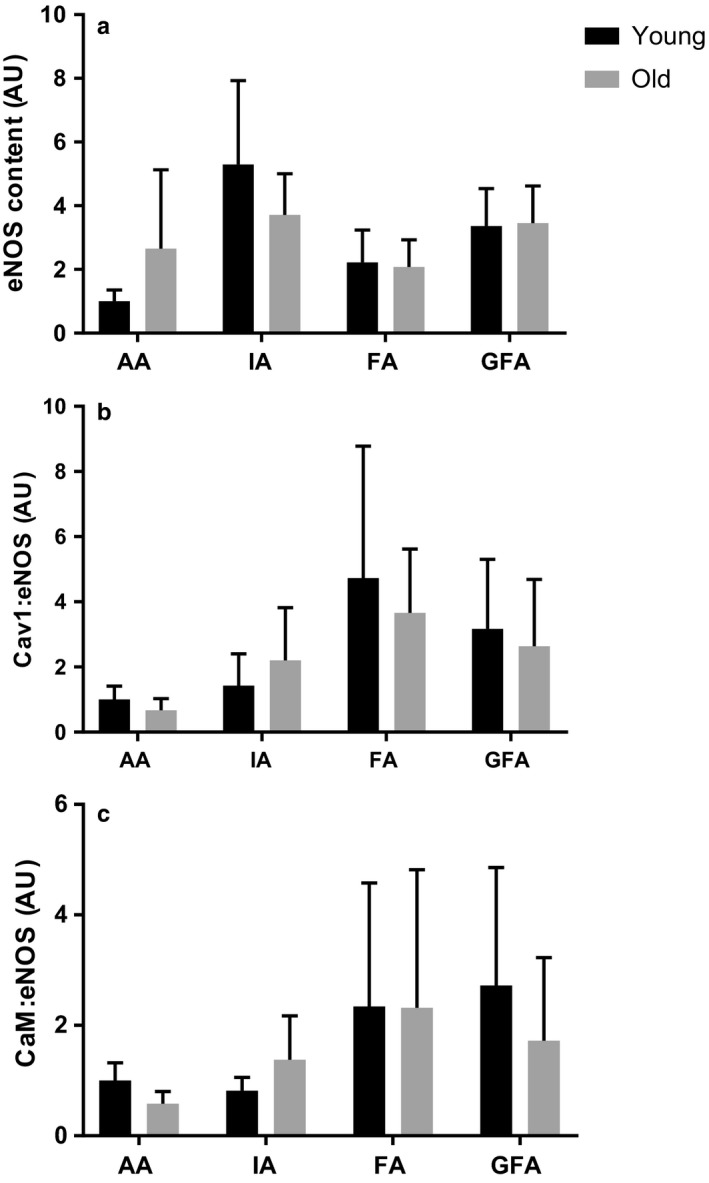
Normalized immunoprecipitated eNOS protein content and protein:protein interactions in arteries from young and old rats. (a) eNOS protein content normalized to eNOS content in young AA. (b) Cav1:eNOS interactions in arteries in young and old rats. (c) CaM:eNOS interactions in arteries in young and old male rats. Values are means ± *SD*; *n* = 7–8 per group

Immunoprecipitated eNOS protein and eNOS‐associated protein:protein interactions in all arteries studied from young and old rats is shown in Figure 3. There were no age‐group differences in normalized immunoprecipitated eNOS protein content in any artery (*p* > .05 for all arteries; Figure 3a). There were also no age‐group differences in either Cav1:eNOS (Figure 3b) or CaM:eNOS (Figure 3c) interactions in any artery studied (*p* > .05 for all arteries).

### 3.4 Pooled total eNOS protein content

**Figure 4:**
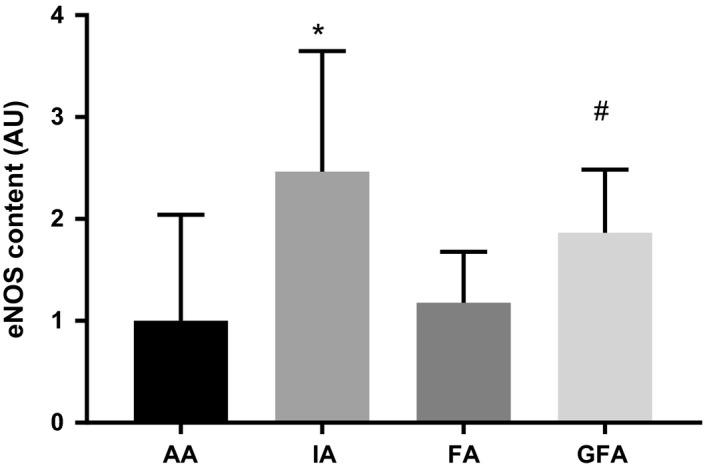
Normalized immunoprecipitated eNOS protein content in large and small arteries. eNOS protein content normalized to eNOS content in AA. Values are means ± *SD*; *n* = 4–6 per group. *Significantly different from AA and FA, ^#^Significantly different from AA, *p* ≤ .05

Normalized immunoprecipitated eNOS protein content in large and small arteries is shown in Figure 4. eNOS protein content was significantly higher in the IA (2.46 ± 1.18 AU) compared to the AA (1 ± 1.04 AU; *p* < .001) and FA (1.18 ± 0.50 AU; *p* < .001). eNOS protein content was also higher in GFA (1.87 ± 0.62 AU) compared to AA (*p* < .05).

### 3.5 Pooled protein:protein interactions

**Figure 5:**
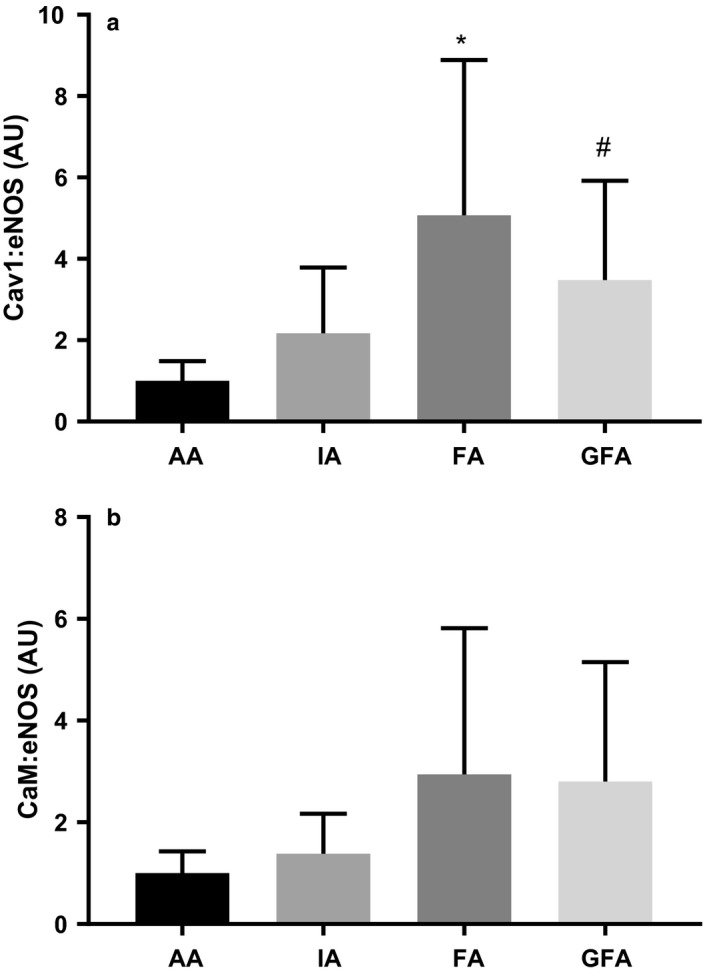
Normalized eNOS‐associated protein:protein interactions in large and small arteries. (a) Cav1:eNOS interactions in large and small arteries. *Significantly different from AA, and IA, ^#^Significantly different from AA, *p* ≤ .05 (b) CaM:eNOS interactions in large and small arteries. Values are means ± *SD*; *n* = 4–6 per group

Cav1:eNOS interaction in large and small arteries is shown in Figure 5a. Cav1:eNOS was significantly higher in the FA (5.07 ± 3.82 AU) compared to the AA (1 ± 0.49 AU; *p* < .01) and IA (2.17 ± 1.62; *p* < .01). Cav1:eNOS was also higher in GFA (3.48 ± 2.446 AU) compared to AA (*p* < .05).

CaM:eNOS interaction in arteries from young and old rats is shown in Figure 5b. Despite a significant main effect (*p* < .05), multiple comparison analysis revealed no significant differences in CaM:eNOS interaction among arteries.

## 4 DISCUSSION

The primary findings from this study are as follows: (1) vasorelaxation responses to ACh are impaired in the large conduit arteries (AA and IA) from old rats, but not in the small conduit (FA) or resistance arteries perfusing glycolytic muscle (GFA); (2) vasorelaxation responses to SNP are impaired in AA only from old rats; (3) immunoprecipicated eNOS protein content and eNOS‐associated protein:protein interactions were not altered by aging; and (4) immunoprecipitated eNOS protein content and Cav1:eNOS interactions are non‐uniformly distributed among large conduit arteries and small conduit and resistance arteries.

Our finding of impaired ACh‐induced vasorelaxation and reduced sensitivity with age in AA, and preserved function in FA and GFA, is consistent with existing literature (Barton et al., 1997; Luttrell et al., 2013; Woodman et al., 2002). Moreover, to our knowledge, this is the first study to investigate the effect of aging on vasorelaxation responses and sensitivity to ACh and SNP in the IA of young and old rats. In our study, the preservation of ACh‐mediated vasorelaxation in the GFA supports and extends the findings of Woodman et al. (2002), who demonstrated preserved flow‐ and ACh‐induced vasorelaxation in GFA from old rats. Woodman and colleagues demonstrated that the preservation of endothelial function in the GFA from older animals was associated with maintained eNOS and superoxide dismutase‐1 (SOD‐1) protein content. Notably, in the soleus feed arteries (SFA) from the same animals, NO‐mediated endothelial function was impaired, and eNOS and SOD‐1 protein content were reduced (Woodman et al., 2002). Our results are also consistent with those of Barton et al. (1997) who demonstrated impaired ACh‐induced relaxation in the aorta, but not the femoral artery, of old compared to young female rats. Barton's work, similar to that of Woodman et al. (2002), also suggests a role for superoxide dismutase (SOD) in the disparate responses of these vessels to aging (Barton et al., 1997). However, it should be noted that the work of Barton et al. (1997) analyzed SOD enzyme activity in the aorta and femoral artery, while Woodman and colleagues measured SOD‐1 protein content in GFA and SFA (Woodman et al., 2002). Regardless, these studies suggest that maintenance of SOD enzyme capacity in the FA and GFA of older rats may help maintain NO bioavailability, and ACh‐mediated endothelial function, in these vessels. Although the role of antioxidant enzyme activity as a potential mechanism accounting for the heterogeneous effects of aging in the arterial system was not the focus of the present study, it remains a viable explanation for the differences in vasorelaxation responses among large and small arteries we observed.

Despite the similarities between our results and those of Woodman et al. (2002 and Barton et al. (1997, there are also discrepancies. First, Woodman and colleagues showed impaired NO‐mediated endothelial function in SFA from aged animals was associated with a reduction in eNOS protein content (Woodman et al., 2002). In our study, we did not observe age‐associated changes in immunoprecipitated eNOS protein content in any artery (Figure 3). Although the co‐immunoprecipitation and immunoblot protocol used in this study is designed to pull down all eNOS protein and associated proteins in each sample, it is possible that differences in the protein isolation technique between our study and others may partially account for discrepancies in age‐associated changes in total eNOS protein between our study and others.

We also observed impaired SNP‐mediated vasorelaxation in AA from older rats. Although this differs from the results of Barton et al. (1997 , our results are consistent with other studies showing attenuated relaxation responses to SNP in aortas from old rats (Delp, Brown, Laughlin, & Hasser, 1995; Miyata et al., 1992; Moritoki et al., 1988; Payne, Reckelhoff, & Khalil, 2003). As Moritoki and colleagues (1988) demonstrate, impaired cyclic GMP signaling within vascular smooth muscle cells from old rats is a likely mechanism accounting for this observation. Consequently, our finding of impaired relaxation responses to ACh in the AA may not reflect impaired NO production by the vascular endothelium, but rather an impaired vascular smooth muscle sensitivity to signals that activate the cyclic GMP signaling cascade. It should also be noted that Barton and colleagues performed their experiments in female animals (Barton et al., 1997), while in our study, and three of the previously cited studies (Delp et al., 1995; Moritoki et al., 1988; Payne et al., 2003) performed experiments in male animals. The sex of the animals used in the study by Miyata and colleagues (1992) was not reported. However, impaired ACh‐ and SNP‐mediated vasorelaxation responses have been demonstrated in aortas of older, ovariectomized female Norwegian brown rats (Stice, Eiserich, & Knowlton, 2009), and in another study, older male and female Wistar rats had a similar decrement in ACh‐ and SNP‐mediated vasorelaxation (Han et al., 2018). At present, the interaction of strain, age, and sex on vasorelaxation function in the AA, IA, FA, and GFA remains unclear.

Previous work in swine provides evidence of heterogeneous eNOS protein expression within the systemic (Simmons, Padilla, & Laughlin, 2012) and coronary (Heaps, Bray, McIntosh, & Schroeder, 2019) vasculature. Our results are consistent with those of Simmons and colleagues, who report similar eNOS expression in the aorta and femoral artery endothelium (Simmons et al., 2012). Moreover, a recent study by Heaps et al. (2019) determined eNOS distribution along the porcine coronary vascular network, and showed a greater eNOS protein content in coronary conduit artery endothelium compared to coronary microvascular endothelium (Heaps et al., 2019). Although our study and that of Simmons et al. (2012) found non‐uniform effect of artery size on eNOS protein content or expression, these studies collectively demonstrate a heterogeneous phenotype of eNOS distribution throughout the systemic and coronary vascular trees. Moreover, to our knowledge, ours is the first study to report eNOS protein content in IA, which we found to be higher than that of either AA or FA. Although the phenotypic heterogeneity of the endothelium is well established (Aird, 2007; Chi et al., 2003), whether the non‐uniform distribution of eNOS in the systemic circulation is a result of developmental programming, different patterns of mechanical and/or metabolic stresses, or a combination of these factors remains unclear.

Our results revealed that aging has no effect on Cav1:eNOS or CaM:eNOS in either large conduit arteries or the smaller arteries (Figure 3). These results are surprising given that we have previously shown an decrease in both eNOS‐associated protein:protein interactions in the aortas of older rats (Luttrell et al., 2013). In addition, work by Smith, Visioli, & Hagen (2006) also showed an increase in Cav1:eNOS interaction in the abdominal aorta with age. This inconsistency suggests that more research to examine and clarify impact of aging on eNOS‐associated protein interactions is warranted.

Our results suggest that eNOS interaction with Cav1 is non‐uniform among large conduit and small conduit and resistance arteries. We show that Cav1:eNOS interaction is higher in the small conduit FA compared to the larger conduit AA and IA. We also found greater Cav1:eNOS interaction in GFA compared to AA. One interpretation of these results may be that Cav1 plays a larger role in regulating eNOS activity in smaller conduit arteries like the FA, compared to larger conduit arteries.

Contrary to our hypothesis, we did not find differences among large and small arteries with respect to CaM:eNOS interaction. Given the role of CaM in relieving Cav1 inhibition of eNOS enzyme activity under conditions that increase endothelial intracellular calcium (Feron, Michel, Sase, & Michel, 1998; Michel et al., 1997), it is possible that, in the unstimulated state of the vessels we analyzed, CaM:eNOS interaction simply reflected basal intracellular calcium concentrations, which may be similar across the vascular endothelium. Regardless, additional research investigating both Cav1 and CaM regulation of eNOS enzyme activity and vasomotor function is warranted.

Clinically, impaired conduit artery function is an independent predictor of cardiovascular disease, and it is thought to precede the development of resistance artery dysfunction (Mitchell, 2008). Data from human studies also suggest that subsequent age‐related impairments NO bioavailability in resistance arteries may alter patterns of oscillatory and retrograde shear stresses on upstream, conduit arteries, (Padilla et al., 2011) which may further exacerbate arterial function in both artery phenotypes. Evidence from other vascular beds suggests that interventions aimed at improving vascular function may have vessel‐specific effects. For example, Walker et al. (2014) have demonstrated greater improvements in endothelial function from the carotid compared to the middle cerebral artery in response to lifelong caloric restriction in mice. Within the coronary vasculature of adult miniature swine, exercise training non‐uniformly alters eNOS protein content (Laughlin et al., 2001), and training also has non‐uniform effects on vascular function in adult rat skeletal muscles (Laughlin, Woodman, Schrage, Gute, & Price, 2004; McAllister, Jasperse, & Laughlin, 2005). These types of interventions may need to be specifically targeted based on the degree of arterial dysfunction present, and on the specific arterial beds that are impacted.

Although the functional importance of eNOS‐associated protein:protein interactions in large conduit, smaller conduit, and resistance‐sized arteries remains unclear, some evidence suggests that the contribution of eNOS‐derived NO to vasorelaxation function is proportional to vessel size, summarized by Shimokawa & Godo (2016). Consequently, greater interaction among eNOS and Cav1 specifically may constrain NO production in smaller vessels, limiting the contribution of NO to vasorelaxation. Data from the present study may be consistent with this, as it suggests that Cav1:eNOS interaction is greater in the smaller vessels (FA, GFA), compared to the AA (Figure 5). While we did not measure eNOS enzyme activity or NO production, the present results suggests that eNOS activity may be constrained in the small conduit FA and resistance‐sized GFA. The importance of CaM:eNOS interactions in conduit versus resistance‐sized arteries is less clear. However, as discussed earlier, the similar level of CaM:eNOS interaction we observed may reflect the basal state of interaction between these two proteins. In the presence of a vasorelaxation stimulus, differences in CaM:eNOS interaction among vessel types may be more apparent.

### 4.1 Limitations

We cannot conclude that the impaired vasorelaxation responses in the AA and IA are due to age‐associated impairments in NO bioavailability, since we did not experimentally inhibit eNOS activity or use a free radical scavenger to eliminate the possibility that high levels of oxidative stress scavenged NO in the vessels from older rats. Thus, we can only conclude that vasorelaxation responses in the AA and IA to ACh and, in the IA, responses to SNP, are impaired with age.

In addition, there are many post‐translational modifications to eNOS that may influence NO production and bioavailability. Here, we only analyzed eNOS‐associated protein interactions with Cav1 and CaM in unstimulated arteries. It is likely that aging alters other post‐translational mechanisms, including eNOS phosphorylation state and cofactor availability in the arteries we studied, and that the effect of aging on these modifications may be heterogeneous throughout the arterial tree.

Finally, functional responses to ACh and SNP were analyzed in one group of rats, whereas the protein content of eNOS and associated Cav1 and CaM were analyzed in arteries from a separate group of rats. Although all rats were from the same genetic background, it is possible that the functional responses and protein level changes with aging may have been different between the two groups of rats.

## 5 CONCLUSION

In conclusion, our results further supports the heterogeneous phenotype of large conduit and small conduit and resistance arteries. We demonstrate a heterogeneous effect of aging in large conduit compared to small conduit and resistance arteries with respect to vasodilator responses to ACh and SNP. We also show molecular heterogeneity within the systemic arterial endothelium with respect to eNOS protein content and Cav1:eNOS interaction. Together, these results suggest that the effect of aging on vasorelaxation function, as well as eNOS‐associated protein interactions, is vessel‐specific.
